# University of California Center for Health Quality and Innovation: experiences from a system approach to scaling up effective interventions

**DOI:** 10.1186/1748-5908-10-S1-A16

**Published:** 2015-08-20

**Authors:** Karyn DiGiorgio, Wendy G Anderson, Maxime Cannesson, Nathaniel Gleason, Edith O'Neill-Page, Michael K Ong

**Affiliations:** 1 Center for Health Quality and Innovation, University of California, Office of the President, Oakland, CA, 94607, USA; 2 Department of Medicine, University of California, San Francisco, San Francisco, CA, 94143, USA; 3 Department of Anesthesiology & Perioperative Care, University of California, Irvine, Orange, CA, 92683, USA; 4 Ronald Reagan UCLA Medical Center, University of California, Los Angeles, CA 90095, USA; 5 Department of Medicine, University of California, Los Angeles, CA 90095, USA

## 

The challenge of improving outcomes, costs, and access in health care among academic health systems is not a dearth of innovative research; but the difficulties encountered when attempting the dissemination and implementation of effective interventions throughout health systems.

The University of California (UC) Center for Health Quality and Innovation (CHQI) was established to mitigate barriers to improvements among UC academic health systems. UC recognizes that engagement by leadership, strong evaluation processes, and innovator champions are key to overcoming dissemination and implementation barriers within large health systems.

CHQI, led by five UC health system CEOs, six medical school deans, and the UC Senior Vice President of Health Sciences and Services, integrates evidence-based interventions by supporting a collaborative approach to innovative research and spreading best practices through grants, leadership, change management training, and convening task forces to facilitate system-wide change. CHQI employs a tactical and methodical performance-monitored approach to disseminating and implementing proven models of care, including quantifying the ROI (in quality and costs) of all programs prior to initiating a spread program. Once the ROI is assessed for scale and feasibility, a team of senior UC researchers provides guidance by reviewing program implementation and process measures with a focus on spreading best practices, as well as ongoing cost and outcome analyses. Since 2011, CHQI has funded 50 innovation projects, and trained and supported 23 fellows who additionally manage their own innovation projects.

This panel presents CHQI's approach and experiences with scaling up effective interventions, through 3 of its multi-site programs that demonstrated a proven ROI before integrating and spreading evidence-based interventions across 5 UC health systems. The panelists will discuss barriers, challenges and successes of project design, evaluation, and implementation, such as data integration issues, organizational barriers (including culture and policies) among health systems, institutional impediments (workflow, workforce), and future plans.

## IMPACT-ICU-integrating multi-disciplinary palliative care into the ICU

One fifth of Americans die after receiving care in an ICU. These patients receive interventions that may not be consistent with their wishes, families experience significant distress, and the costs of unwanted care burden our health system. When integrated into ICUs, palliative care improves management of patients' symptoms, decreases family distress, and increases satisfaction. It also decreases ICU length of stay and costs. The IMPACT-ICU is a quality improvement program to increase integration of palliative care into the ICU by educating ICU nurses to identify and address needs in 3 domains: 1) patient symptom management, 2) family support, and 3) multidisciplinary communication about prognosis and goals of care. A pilot program at UCSF improved ICU nurse confidence and skills in palliative care discussions, and increased palliative care consults from the ICU. The objective of this project is to increase the integration of palliative care in the ICUs at the 5 UC medical centers. This objective is accomplished through a multidisciplinary collaborative of ICU and palliative care nurse and physician leaders from the 5 medical centers. The collaborative achieves two aims: 1) Expand a training program to increase the involvement of ICU nurses in communication about prognosis, goals of care and palliative care for seriously ill patients, 2) Identify best practices in ICU-palliative care integration and implement them to complement and support the nurse education intervention. This program has now trained 250 ICU nurses and has again demonstrated improvements in ICU nurse confidence and skills in palliative care discussions.

## Dissemination of enhanced recovery after surgery (ERAS) toolbox for high risk surgery patients

Every year about 240 million surgical procedures are performed globally. For the UC system alone, 110,000 patients undergo surgery each year. While high-risk surgery procedures represent only about 12.5 % of this surgical volume, they account for about 80% of overall patient mortality related to surgery. In addition, the incidence of postoperative complications in patients undergoing high-risk surgical procedures is about 30%. As such, there is urgent need to develop and adopt interventions that are direct at improving the outcomes of high-risk surgical procedures. Enhanced Recovery After Surgery (ERAS) is a bundle of best evidence based practices aimed at enhancing patient postoperative recovery and outcomes following high-risk surgery. This innovative program includes management of perioperative pain, nausea and vomiting, transfusion, and goal directed fluid administration and hemodynamic optimization. Where ERAS is embedded, participating sites report improved patient experience, clinical outcomes, and multi-disciplinary team collaboration and reduction in length of stay and risk of hospital acquired infections. The UCI Enhanced Recovery After Surgery (ERAS) program for patients undergoing high-risk abdominal surgery patients demonstrated a reduction in length of stay by 2 days. A "Toolbox" for the dissemination of this program system-wide includes online pre-tests and post-tests, online training, written protocols, handouts for Goal Directed Therapy application at the bedside, and documents explaining the key factors for success and the barriers to implementation and how to overcome them. This Toolbox is currently being disseminated to all UC medical centers for patients undergoing high-risk abdominal, gynecologic, urological, and orthopedic surgeries.

## e-Referrals and e-Consults

The UCSF e-Consult program allowed Primary Care Providers to receive timely, low-cost input from specialists on lower-complexity and data-oriented clinical questions that do not require an in-person evaluation.

## Impact

One year after its launch, the UCSF experience demonstrated significant impact on referral rate, specialty care utilization, specialty care access time, and costs. eConsults now represent 8.2% of referrals to participating specialties. The referral rate for standard office visits declined by 20%. Access to a specialty care input within 14-days (via e-Consult or office visit) improved from 29% to 46%, a 59% improvement. Mean professional-fees during the 120-day period following all referrals or e-Consults decreased by 7.2%. Adoption of the program is robust, with 2/3 PCPs using e-Consult, and high acceptability among providers. Conservative modeling yields an anticipated savings of $250,000 annually per 50,000 primary care patients. Program costs for that same population will be approximately $45,000 per year for e-Consult fees (to be replaced ultimately by payer reimbursement), as well as site-leader and site-analyst support, with personnel cost dropping sharply after the first year. The UCSF e-Consult program is now in the process of being spread to the other 4 UC medical centers to further drive integration of primary care and specialty care-an essential step to deliver higher value care. Some initial factors that this spread effort has had to account for are the differences in electronic health record systems among the UC medical centers.

